# Supplementary material for: Adjusted trajectory of medication exposure taking into account the periodicity of dispensations and the number of dispensed packs and comparative analysis on EFEMERIS database
Source: PLoS One. 2025 Feb 13;20(2):e0308767. doi: 10.1371/journal.pone.0308767 (PMC11824950; doi:10.1371/journal.pone.0308767)
Supplement: Attachment — Submitted filename: Response to Reviewers.pdf [file pone.0308767.s001.pdf]

Toulouse Mathematics Institute
University Paul Sabatier Toulouse 3
118 route de Narbonne
31400 Toulouse, France

December 5, 2024

Dear Editor and dear reviewers:

We wish to submit our revised manuscript of our research article for publication in PLOS ONE, titled "Adjusted trajectory of medication exposure taking into account the periodicity of dispensations and the number of dispensed packs and comparative analysis on EFEMERIS database" (coauthored by Cécile Chouquet, Anna-Belle Beau, Christine Damase-Michel, David Jeauneau, Isabelle Lacroix and Sabine Mercier).

First of all, we thank you for your answers and your relevant remarks allowing us to clarify and improve the manuscript. Below we respond to the comments of the academic editor and the reviewers on each point requiring a response after recalling them in italics.

Answers to the academic editor

Point 2. We note that you have indicated that there are restrictions to data sharing for this study. For studies involving human research participant data or other sensitive data, we encourage authors to share de-identified or anonymized data. However, when data cannot be publicly shared for ethical reasons, we allow authors to make their data sets available upon request.

Concerning data sharing: data cannot be publicly shared for ethical and legal restrictions. Indeed EFEMERIS data is pseudonymized. Data are not directly identifying but are not appropriate to share because:

- It concerns sensitive health data (medication exposure, and children pathologies).
- In some analyses, the number of pregnant women is less than 5, which makes the data potentially identifiable.
- The Commission Nationale de l'Informatique et des Libertés (CNIL) does not authorize us to share EFEMERIS data.

To confirm the non-authorization of access to data, the contact is the Data protection officer of the CHU of Toulouse (Dpo@chu-toulouse.fr).

Point 3. Please note that your Data Availability Statement is currently missing [the repository name and/or the DOI/accession number of each dataset OR a direct link to access each database]. If your manuscript is accepted for publication, you will be asked to provide these details on a very short timeline. We therefore suggest that you provide this information now, though we will not hold up the peer review process if you are unable.

Given the restrictions on sharing data from the EFEMERIS cohort, point 3 does not concern us.

Point 4. Please review your reference list to ensure that it is complete and correct. If you have cited papers that have been retracted, please include the rationale for doing so in the manuscript text, or remove these references and replace them with relevant current references. Any changes to the reference list should be mentioned in the rebuttal letter that accompanies your revised manuscript. If you need to cite a retracted article, indicate the article's retracted status in the References list and also include a citation and full reference for the retraction notice.

The list of references has been checked, reviewed and expanded. We have also made some modifications to address the comments 1 and 2 of reviewer 1 (see below).

Answers to the review Comments

Reviewer 1

Comment 1. Although throughout results and discussion section, results are adequately discussed, authors did not provide sufficient number of references of recent studies to support and explain the obtained data.
Comment 2. Isabelle Lacroix appears as a very high percentage in the list of references. We suggest that this percentage be reduced so that it is not considered excessive self-citation.

Answer to comments 1 and 2:
The Section Introduction has been revised to take into account additional references on methods recently developed and/or used to model drug exposure over time and to group trajectories into exposure profiles (cf. ref. [4-8]). Furthermore, we kept only one of the 2 references presenting EFEMERIS in Section Data to balance the number of references between those attributable to the authors of our article, and those of other research teams. In the same section, we added one reference on the representativeness of the EFEMERIS database at the national level (cf. added ref. [12]). The number of references has therefore increased from 7 to 14 references, including 4 citing one of the authors of our article (compared to 5 out of 7 in the previous version).

Reviewer 2

The data come from a specific area (Haute-Garonne, France), and it would be helpful for the authors to address more broadly the potential limitations of generalizing the results to other populations.

Answer:
We responded to the remarks about the generalizability of the findings from two points of view: one concerning the representativeness of EFEMERIS data, and the other the generalization of the method with regard to the information provided in the databases.

- The first concerns the EFEMERIS database whose women studied come from Haute-Garonne. A study of the representativeness of this database was carried out by Demailly *et al.* (cf. added ref. [12]) on the prescription and dispensing data of drugs in pregnant women. We added this element in the Section Data (lines 90-91) and cited this reference.
- We also responded to your comment from an angle relating to the generalization of the method itself and its use on other databases. For this purpose, we have added a paragraph (lines 70-75) at the end of the Subsection "Proposed method ... periodicity adjustment" to mention the necessary information for the application of the method: namely the name and ATC of the dispensed drug, the number of dispensed packs, and the date of dispensing. Such data are commonly available in European Health care data sources as mentioned by Charlton (cf. added ref. [11]).

The purpose of the article is to present the method. The EFEMERIS database is used as example to illustrate the method. The pharmaco-epidemiological developed results themselves will be the subject of other articles currently being written.

Discussion on the impact of classification changes shows that only 4% of trajectories change clusters, so a more in-depth discussion of these changes and their impact on the results could be useful.

Answer:
Step 3 of Section Results and discussion has been totally modified. We integrated two new tables.

- The first one (Table 2) corresponds to a concordance table between the clusters of scenarios 2 and 3 (see Subsubsection "Concordance between scenarios 2 and 3") to clarify and better understand the changes in partition. We commented on the main changes in numbers of trajectories by cluster. We then highlighted what

we call major changes meaning, non-exposed trajectory becoming exposed or vice versa, or trajectories changing in at least two exposure cluster levels.

- The second one (Table 3) presents in Subsubsection "Comparison of weekly DDD values and neonatal pathology by cluster between scenarios 2 and 3", classical statistical indicators for each cluster and each scenario to see how the content of the four clusters changes. We discuss the main changes in particular for the cluster D and the impact on the neonatal pathology.

More targeted analyses to model neonatal risk of disease based on exposure clusters (such as logistic regression or other) will be the subject of other publications.

All changes (those mentioned above and other more minor ones) made to the original version were highlighted in the marked-up copy of our manuscript. Please note that we have also modified and completed the Human Subjects Research Checklist.

We thank you again for your feedback and hope to have responded fully.

Thank you for your consideration.

Sincerely,

Cécile Chouquet
